# The Expression of HPV-16 E5 Oncoprotein Impacts the Transcript Profiles of FGFR2 and EMT-Related Genes in Preneoplastic Anal Epithelium Lesions

**DOI:** 10.3390/ijms252212085

**Published:** 2024-11-11

**Authors:** Salvatore Raffa, Vanessa Mancini, Deborah French, Francesca Rollo, Maria Benevolo, Eugenia Giuliani, Maria Gabriella Donà, Danilo Ranieri, Francesca Belleudi

**Affiliations:** 1 Department of Clinical and Molecular Medicine, Sapienza University, 00189 Rome, Italy; salvatore.raffa@uniroma1.it (S.R.); vanessa.mancini@uniroma1.it (V.M.); deborah.french@uniroma1.it (D.F.); francesca.belleudi@uniroma1.it (F.B.); 2 Medical Genetics and Advanced Cellular Diagnostics Unit and Cross-Departmental Program for Integrated Diagnosis of HPV-Related Diseases, Sant’Andrea University Hospital, 00189 Rome, Italy; 3 Pathology Department, IRCCS Regina Elena National Cancer Institute, 00144 Rome, Italy; francesca.rollo@ifo.it (F.R.); maria.benevolo@ifo.it (M.B.); 4 Sexually Transmitted Infection/Human Immunodeficiency Virus Unit, San Gallicano Dermatological Institute IRCCS, 00144 Rome, Italy; eugenia.giuliani@ifo.it (E.G.); mariagabriella.dona@ifo.it (M.G.D.); 5 Department of Life Sciences, Health and Health Professions, Link Campus University, 00165 Rome, Italy

**Keywords:** E5, HPV16, FGFR2c, epithelial–mesenchymal transition, TRPA1

## Abstract

Anal Squamous Cell Carcinoma (SCCA) is a rare Human Papillomavirus type 16 (HPV16)-associated carcinoma whose pathogenesis is still poorly understood. Recent studies based on biopsy and Next Generation Sequencing (NGS) approaches have linked the viral episomal status to aggressive SCCA phenotypes, suggesting a potential role of the 16E5 oncoprotein in tumor development. Our previous findings indicated that 16E5 induces Fibroblast Growth Factor Receptor 2 (FGFR2) isoform switching, aberrant mesenchymal FGFR2c expression, Epithelial Mesenchymal Transition (EMT), and cell invasion in various in vitro human keratinocyte models, as well as in the in vivo context of cervical Low-grade Squamous Intraepithelial Lesions (LSILs). To further explore the role of 16E5 in epithelial carcinogenesis, this study aims to investigate the molecular profile in HPV-related anal lesions. The results showed a significant positive correlation between 16E5 and FGFR2c, as well as 16E5 or FGFR2c and key EMT-related transcription factors, particularly in the group of HPV16 positive anal samples not containing without high grade lesions. Additionally, by coupling the molecular analysis with an interactome investigation, we hypothesized a potential functional interplay between the Ca^2+^ channel Transient Receptor Potential Ankyrin 1 (TRPA1) and FGFR2c, mediated by 16E5 during the establishment of the oncogenic signaling. These findings will help to elucidate the actual relevance of 16E5 in the early progression of anal lesions and contribute to determine its potential as target for future preventive approaches for HPV16-positive SCCA.

## 1. Introduction

Among the high-risk cancer-causing HPVs, HPV16 and HPV18 are considered the most tumorigenic, associated with up to 70% of cases of the invasive cervical cancers in the world [1,2] and to other neoplasia, such as head and neck (HNSCC) and anogenital tumors [2]. Even if E6 and E7 are the viral early proteins playing the main oncogenic role [2,3], the evidence of HPV-associated cancers containing a mixture of integrate/episomal forms or exclusively episomes of HPV [4,5,6], where the expression of E5 is maintained [7,8,9], have suggested that a more relevant function in carcinogenesis could be played also by other early proteins, including E5 [10]. In agreement with this possibility, our previous data demonstrated that the expression of the E5 protein of HPV16 (16E5) alone is able to perturb epidermal homeostasis driving fibroblast growth factor receptor 2 (FGFR2) isoform switch and consequent aberrant expression of the mesenchymal FGFR2c variant, which leads to EMT and invasive behavior [11,12,13]. More recently, data obtained coupling in vitro approaches to the molecular analysis of transcript expression in cervical low-grade lesions (LSILs) further strengthened the pivotal role of 16E5, demonstrating a direct correlation between 16E5 expression, FGFR2c, and the development of an EMT-associated molecular profile in LSILs [14].

Anal squamous cell carcinoma (SCCA) is another HPV-related cancer that can arise from the progression of intraepithelial lesions, for which the same cervical grading system of LSIL and high-grade Squamous Intraepithelial Lesions (HSIL) lesions is applied [15]. The incidence of this rare neoplasia has significantly risen [16,17], especially in men who have sex with men (MSM). HPV-16 is involved in over 70% of cases [15,18], but the role played by HPV and by its early proteins in SCCA development remain still poorly understood, possibly due to the limited availability of relevant in vitro models. Interestingly, a recent retrospective study demonstrating a unique episomal status of HPV16 in both primary tumor and metastasis in a clinical case of high-grade metastatic SCCA, revealed that even in anal cancer the expression of 16E5 could contribute to malignant progression [19]. This evidence, coupled with the detection of E5 in several HPV16-positive anal cancer biopsies [20], strongly encouraged us to further investigate the role played by this viral protein during the progression of HPV16-positive dysplasia to high grade lesion, analyzing the possible correlation between the 16E5 amount and the appearance of FGFR2c, as well as the acquisition of an EMT signature.

Overall, our study, contributing to the identification of additional oncogenic players and signaling pathways whose dysregulation could be ascribed to 16E5, could help to further strengthen the hypothesis of a pivotal role of this viral oncoprotein particularly in the early steps of HPV16-associated epithelial cancerogenesis.

## 2. Results

### 2.1. 16E5 and FGFR2c Expression in Anal Intraepithelial Lesions

Previous work in our group demonstrated that 16E5 alone is able to induce an FGFR2 isoform switch in normal epidermal keratinocytes [12], while molecular data from cervical LSIL samples revealed a significant positive correlation between the expression of 16E5 and FGFR2c [14]. Intriguingly, an “in vitro” model of human keratinocytes stably expressing FGFR2c, while maintaining unaltered the expression of endogenous epithelial FGFR2b isoform, highlighted that the appearance of the mesenchymal variant, rather than the receptor isoform switch, is the key event required for the acquisition of tumorigenic traits [13].

**Figure 1:**
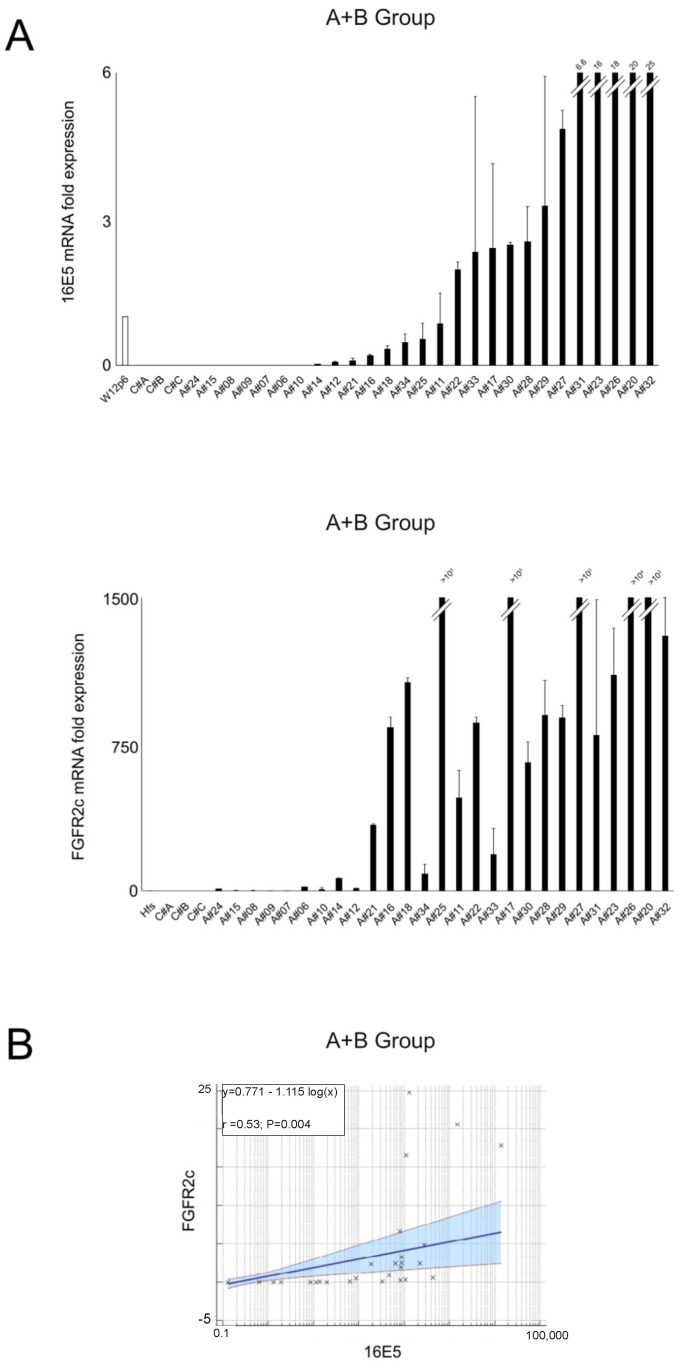
(**A**) Expression levels of HPV16E5 and FGFR2 isoforms in all samples placed in ascending order of expression of 16E5; the 16E5 and FGFR2c mRNA levels were normalized with respect to W12p6 cells or HFs cells (mean values ± SD). (**B**) Relationships between HPV16E5 and FGFR2c expression levels (R^2^: Pearson’s correlation coefficient value; the regression line ±95% confidence limits and ±95% prediction bands are presented in blue and in red, respectively).

To investigate whether a link between FGFR2c appearance and 16E5 expression could exist also in the in vivo pathological context of HPV16-positive anal intraepithelial lesions, the mRNA levels of both 16E5 and FGFR2c were measured in a group of HPV16-positive anal samples (n = 27; A+B group, see explanation in Section 4) using real-time RT-PCR. For 16E5 mRNA normalization, the W12 cervical keratinocyte cell line derived from LSIL was used at a passage previously tested for the presence of about 100–200 copies of the HPV16 episomes (W12p6) [21]. Anal samples negative for the presence of HPV16 were used as negative controls. When samples were sorted based on increasing 16E5 mRNA levels, we found that the FGFR2c transcript levels displayed an increasing trend overlapping that of 16E5 (Figure 1A). Notably, all the HPV16-positive samples showed higher levels of FGFR2c compared to human fibroblast cultures (HFs), a positive control for FGFR2c expression. Conversely, all HPV16-negative controls displayed undetectable FGFR2c mRNA levels (Figure 1A). Statistical analysis confirmed a significant positive correlation between FGFR2c and 16E5 expression (Figure 1B).

**Figure 2:**
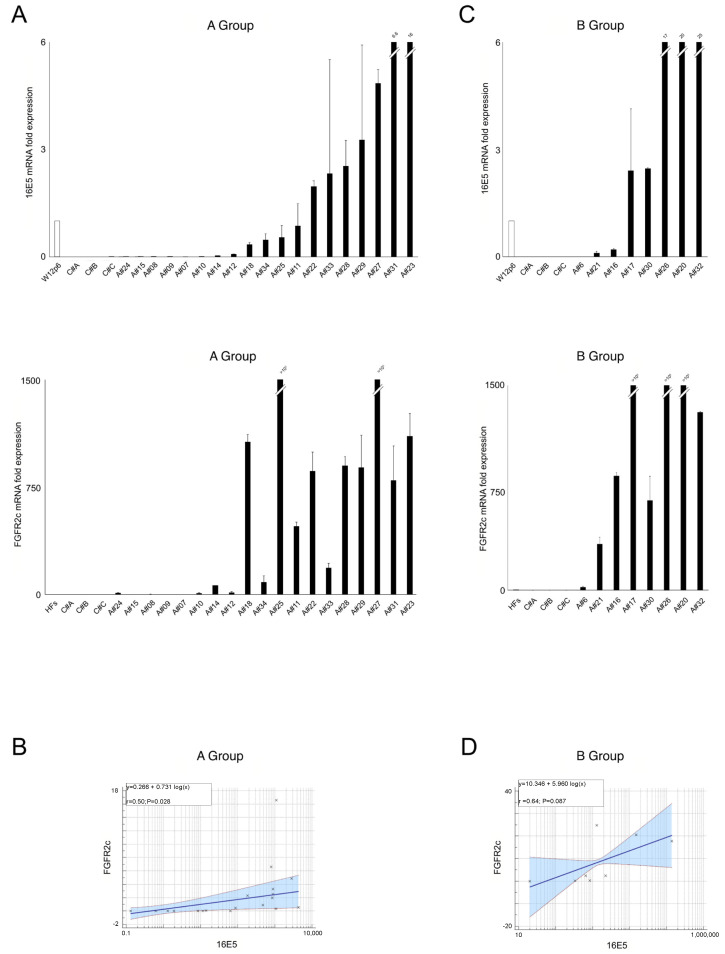
(**A**,**C**) Expression levels of HPV16E5 and FGFR2 isoforms in groups A and B (see Section 4) placed in ascending order of expression of 16E5; the 16E5 and FGFR2c mRNA levels were normalized with respect to W12p6 cells or HFs cells (mean values ± SD). (**B**,**D**) Relationships between HPV16E5 and FGFR2c expression levels (R^2^: Pearson’s correlation coefficient value; the regression line ±95% confidence limits and ±95% prediction bands are presented in blue and in red, respectively).

In a second step, we categorized samples based on their cytopathologic features. Eight samples classified as high grade squamous intraepithelial lesions (HSILs) were grouped into group B, while the remaining HPV16-positive samples, not categorized as high-grade lesions, were placed in group A as reported in the Section 4. We found a direct association between the FGFR2c and 16E5 expression in both group A and group B samples (Figure 2A,C), with significant correlation levels in the A samples (Figure 2B) and a trend toward statistical significance in the B samples (Figure 2D). Interestingly, the very low levels of both 16E5 and FGFR2c mRNAs in the five samples negative for intraepithelial lesion or malignancy (NILM) of group A (#A8, #A10, #A14, #A15, and #A24) were consistent with the hypothesis that the 16E5 impact on FGFR2c expression coincides with the onset of dysplasia.

### 2.2. EMT-Related Transcription Factors and Their Association with 16E5 and FGFR2c Expression

Previous studies of our group have demonstrated that the 16E5-mediated FGFR2 isoform switch is linked to EMT [12] suggesting that the specific induction of FGFR2c is the underlying molecular mechanism [13]. Our recent molecular investigation, performed in the in vivo context of cervical low-grade lesions, further supports the hypothesis of a direct link between 16E5 and the EMT program, possibly through the induction of FGFR2c [14].

Therefore, in this work we attempted to assess whether a link between 16E5, FGFR2c, and the EMT program also exists in the context of the anal cytological abnormalities, monitoring the induction of master EMT-related transcription factors, such as Snail1, Snail2, and ZEB1. In fact, it is well-known that these growth factors are responsible for the downstream repression of epithelial markers and the induction of mesenchymal ones [22].

**Figure 3:**
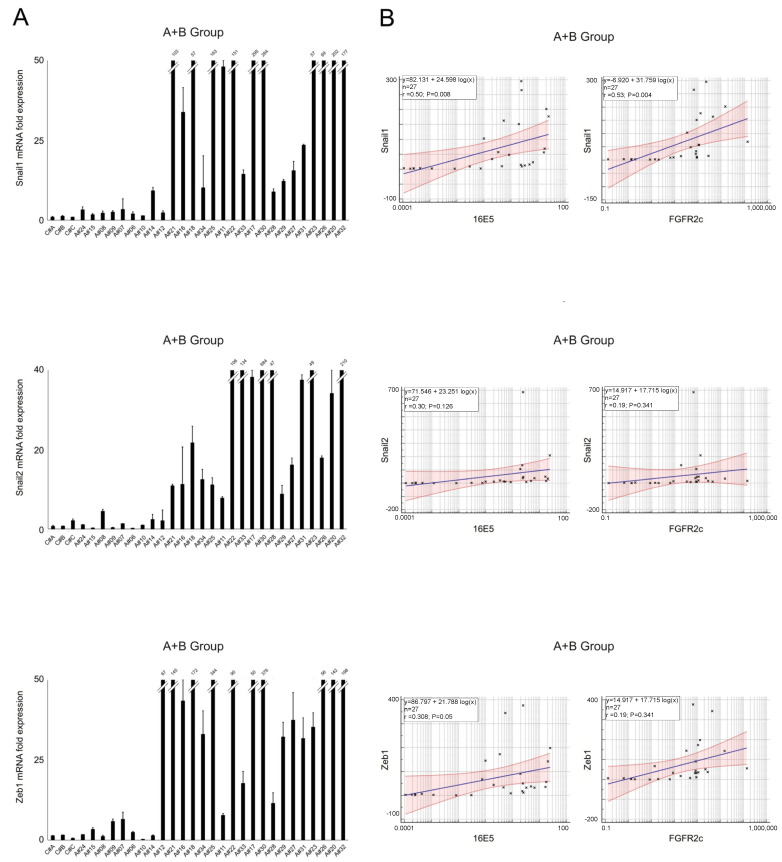
(**A**) Expression levels of EMT-related transcription factors Snail1, Snail2, and ZEB1 in all samples (mean values ± SD). (**B**) Relationships between EMT-related transcription factors regarding HPV16E5 and FGFR2c expression levels (R^2^: Pearson’s correlation coefficient value; the regression line ±95% confidence limits and ±95% prediction bands are presented in blue and red, respectively).

Molecular analysis of the total samples (A+B group) revealed that, while the expression levels of all analyzed transcription factors displayed high variability (Figure 3A), not always aligning with the trend of 16E5 expression (see Figure 1A), a positive correlation was observed between each of these transcription factors and 16E5, becoming significant for Snail1 and ZEB1 (Figure 3B). Interestingly, lower expression levels of Snail1, Snail2, and ZEB1 transcripts were detected not only in 16E5-negative controls (C#) but also in HPV16-positive samples expressing lower levels of 16E5 mRNA (from A#24 to A#14) (Figure 3A). Statistical analysis also demonstrated a significant positive correlation between the Snail1 and ZEB1 transcription factors and FGFR2c (Figure 3B).

**Figure 4:**
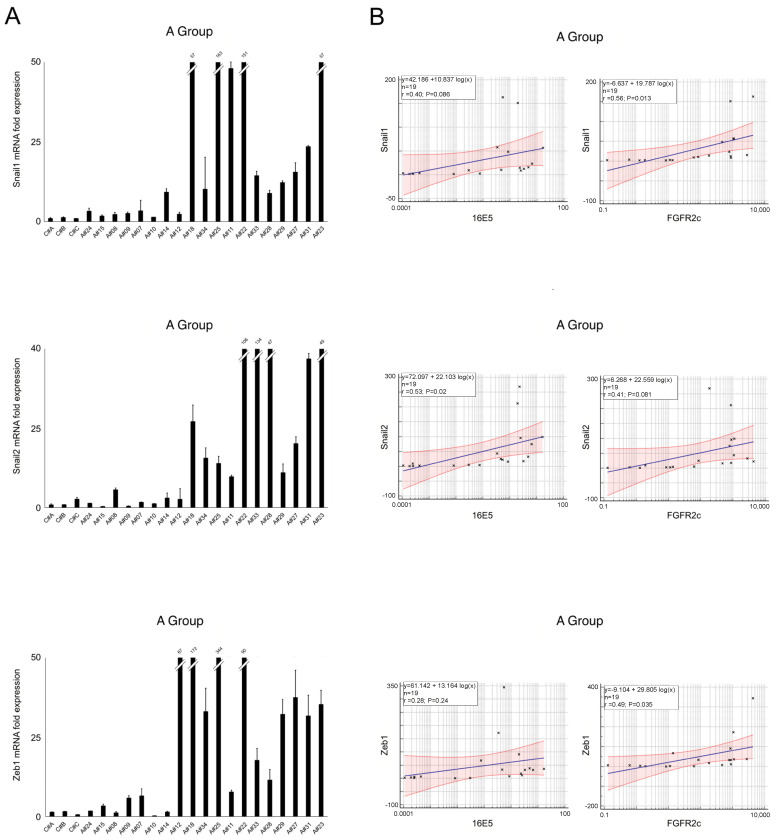
(**A**) Expression levels of EMT-related transcription factors Snail1, Snail2, and ZEB1 in group A samples. (**B**) Relationships between EMT-related transcription factors regarding HPV16E5 and FGFR2c expression levels (R^2^: Pearson’s correlation coefficient value; the regression line ±95% confidence limits and ±95% prediction bands are presented in blue and red, respectively).

**Figure 5:**
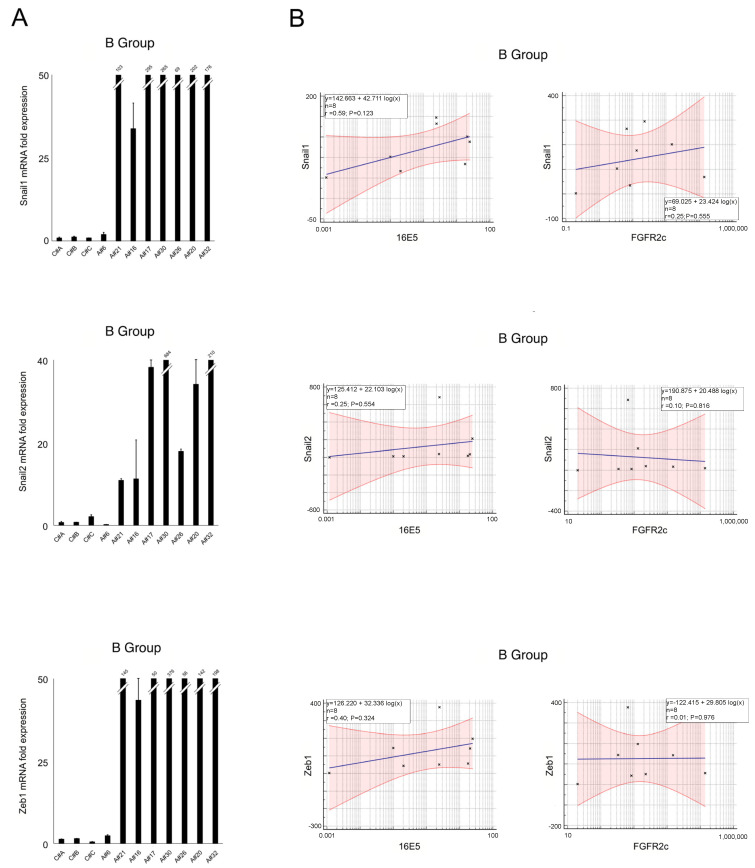
(**A**) Expression levels of EMT-related transcription factors Snail1, Snail2, and ZEB1 in group B samples (mean values ± SD). (**B**) Relationships between EMT-related transcription factors regarding HPV16E5 and FGFR2c expression levels (R^2^: Pearson’s correlation coefficient value; the regression line ±95% confidence limits and ±95% prediction bands are presented in blue and in red, respectively).

Molecular analysis of the A group revealed heterogeneity of the EMT-related genes, as well as their positive correlation with both 16E5 and FGFR2c, which persisted at least for Snail1 and ZEB1 (Figure 4A,B). Conversely, samples in the B group displayed higher and more homogeneous expression of Snail1 and ZEB1 (Figure 5A), with poor overlap with 16E5 and/or FGFR2c mRNA trends (Figure 1A) and a loss of a statistically significant correlation (Figure 5B).

### 2.3. TRPA1 Expression and Its Relationship with 16E5 and FGFR2c in Anal Lesions

**Figure 6:**
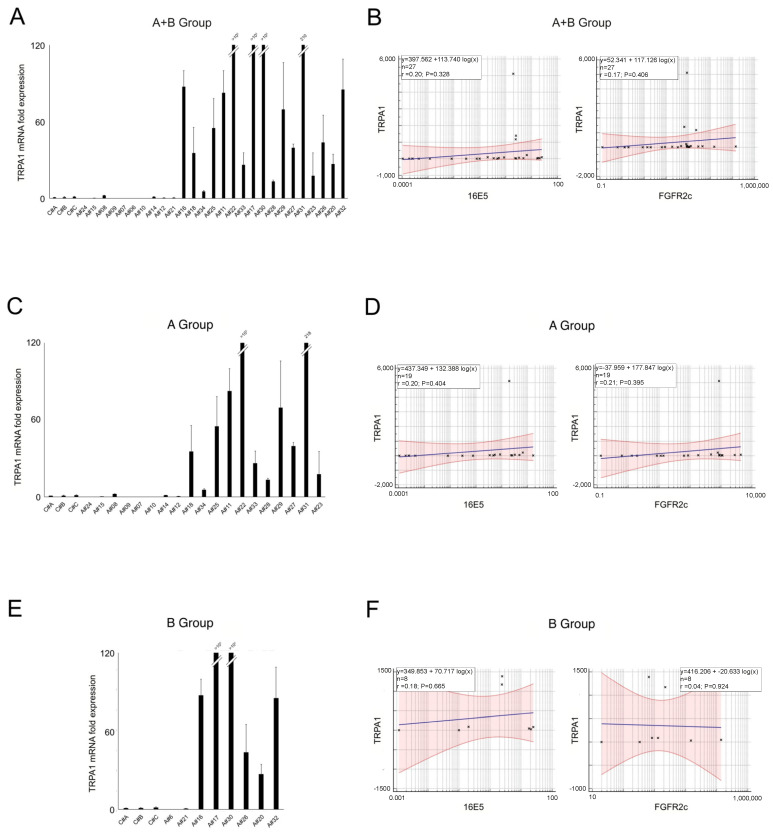
Expression levels of TRPA1 in all samples (**A**) and in groups A (**C**) or B (**E**); mean values ± SD). (**B**,**D**,**F**) Relationships between TRPA1 and the HPV16E5 and FGFR2c expression levels (R^2^: Pearson’s correlation coefficient value; the regression line ±95% confidence limits and ±95% prediction bands are presented in blue and in red, respectively).

To identify additional molecular players potentially contributing to 16E5/FGFR2c-driven anal tumorigenesis, we focused on the Ca^2+^-specific transient receptor potential channel 1 (TRPA1). In fact, our previous research suggested that this channel plays a significant role in the establishment of FGFR2c-mediated aberrant signaling, leading to an enhancement in the EMT phenotype and in the invasive behavior in pancreatic ductal adenocarcinoma cell lines [23]. Real-time RT-PCR analysis performed in all samples (A+B group) revealed very low TRPA1 expression in both the negative controls and HPV16-positive samples with low 16E5 levels (from A#24 to A#14, see Figure 1A). On the other hand, in samples with progressively higher levels of 16E5, the TRPA1 mRNA levels appeared to increase, but these were also highly variable (Figure 6A), and the statistical analysis revealed no significant correlation with either 16E5 and FGFR2c (Figure 6B). Similar results were observed when analyzing the groups separately. In both cases, heterogeneous TRPA1 expression was detected in the 16E5-positive samples (Figure 6C,E), and no significant correlation with the expression of the viral protein or FGFR2c was found (Figure 6D,F).

These findings suggest that TRPA1 protein expression may not be controlled by 16E5 at the transcriptional level. However, this molecular analysis did not rule out the possibility of a post-translational role of 16E5 in regulating TRPA1 functions.

**Figure 7:**
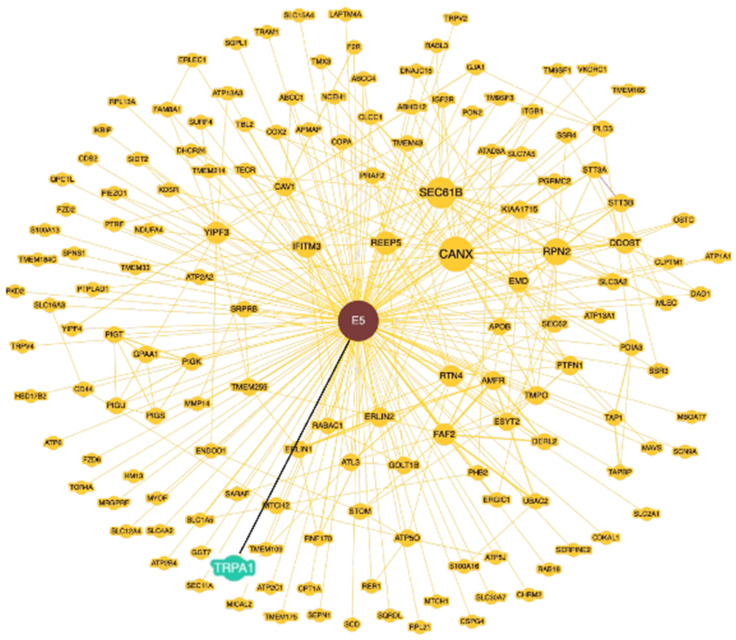
The BioGRID database of proteins that interact with 16E5. The Network module shows 153 proteins interaction, and among these highlighted in blue and circled in red, we find TRPA1, with which a physical interaction is indicated.

To explore this hypothesis, we utilized the Biological General Repository for Interaction Datasets (BioGRID) (https://thebiogrid.org/ (accessed on 10 June 2024)) to perform a 16E5/mammalian protein interactome elaboration. This freely available database provides protein and genetic interactions from multiple species [24]. Using the database’s “Network” module, we generated a 16E5 interactome graph that showed 152 interacting proteins (Figure 7), including TRPA1, whose physical interaction with 16E5 has been previously reported by Rozenblatt-Rosen and co-workers [25]. Thus, the possibility of a functional link between 16E5 and TRPA1 cannot be excluded and would be worth investigating in the future, in the context of precocious anal lesions.

## 3. Discussion

Despite the ever-wider use of HPV vaccines, the incidence of SCCA, a poorly-spread HPV-associated neoplasia, has risen in recent years [16,17], particularly among MSM [15,18]. This is also due to the difficulty in detecting anal precocious and pre-neoplastic lesions, compared to cervical dysplasia.

Unfortunately, the commonly used combined therapies for SCCA management, which couple chemotherapy, radiation, immunotherapy, and target therapy [16], often result in treatment-related toxicity [17]. This highlights an urgent need to identify new molecular targets and oncogenic signaling pathways for more advanced and personalized therapeutic strategies. In total, 76–94% of SCCAs can be attributed to HPV16 virus infection [15,16,18,26,27,28], leading to ongoing research exploring the use of HPV16-derived biomarkers for pre-neoplastic and SCCA screening [16]. However, despite this well-established association, the molecular mechanisms underlying the pre-neoplastic progression remain poorly understood, as is the role played by the virus and its early proteins.

Therefore, aim of this study has been to advance the knowledge of the potential role played by 16E5 in anal dysplasia progression, particularly in relation to its previously demonstrated ability to perturb FGFR2 signaling, leading to oncogenic drift in other epithelial contexts [11,12,13,14]. Our previous findings are further supported by a study demonstrating that, in HPV episome-associated cervical and HNSCC cancers lacking E6/E7 oncoprotein expression, high 16E5 expression is linked to dysregulated cell growth and invasion through the amplification of FGFR signaling. This suggests that 16E5 plays a pivotal role in HPV-associated cancerogenesis, by dysregulating the FGF/FGFR axis [29].

To address our topic, we first evaluated whether a potential link between the expression of 16E5 and FGFR2c exists in anal lesions. Molecular analysis using real-time RT-PCR revealed a significant positive correlation between the FGFR2c and 16E5 mRNA levels. To determine whether the correlation between 16E5 and FGFR2c was dependent on the stage of lesion progression, we separately analyzed HSIL (group B) and non-HSIL (group A) HPV16-positive samples. In both groups, we observed a positive correlation between 16E5 and FGFR2c expression, which remained statistically significant in group A.

To understand whether these expression profiles can contribute to anal lesion progression, we assessed the EMT signature by analyzing the expression of key EMT-related transcription factors (Snail1, Snail2, and ZEB1). In fact, we recently proposed a possible link between 16E5, FGFR2c induction, and the EMT program in in vivo cervical pre-neoplastic lesions [14]. The molecular analysis of all HPV16-positive samples revealed a high degree of variability in mRNA expression for all genes studied. However, a significant positive correlation was observed between Snail1 and ZEB1 with both 16E5 and FGFR2c, even in the non-HSIL group. In contrast, the HSILs showed higher and more homogeneous expression of Snail1 and ZEB1 but lacked a significant correlation with 16E5 or FGFR2c. These results suggest the idea that 16E5 could mainly act in the early steps of dysplasia by modulating EMT-related transcription factors, possibly through the induction of FGFR2c expression and signaling. In contrast, despite the phenotypic heterogeneity of the HSIL samples frequently characterized by the presence of LSIL cells, our data suggest that, when the lesions progress towards the HSIL status, the EMT program is already established, becoming independent from 16E5 and FGFR2 expression.

Finally, looking for other molecular players potentially involved in 16E5-mediated oncogenic progression, we focused on the Ca^2+^ channel TRPA1. Our previous research demonstrated that TRPA1 plays a significant role in the FGFR2c-mediated enhancement of EMT and invasion in the context of pancreatic adenocarcinoma [23]. However, molecular analysis revealed no correlation between the TRPA1 mRNA levels and those of 16E5 or FGFR2c, whether considering all the lesions in a single group or separating them by the cytological report. Thus, 16E5 does not appear to transcriptionally control TRPA1. Nevertheless, interactome analysis identified TRPA1 as one of the mammalian proteins able to bind 16E5, suggesting the possibility of a functional interplay between them. Therefore, it could be speculated that 16E5 may not only control the induction of FGFR2c but also regulate its oncogenic signaling by functionally modulating the receptor crosstalk with membrane partners, such as TRPA1.

Overall, contributing to the identification of additional oncogenic players dysregulated by 16E5, our study could help to define its actual relevance in anal dysplasia progression. Our results may also allow us to assess whether it is worth considering this viral oncoprotein and its cellular targets as future candidates for new target strategies in prevention approaches for HPV16-associated SCCA.

## 4. Materials and Methods

### 4.1. Etichs Statement

This study was conducted in accordance with the Declaration of Helsinki and was approved by the Regional Ethics Committee “Comitato Etico Territoriale Lazio Area 5” (161/IRE/24) on 4 June 2024 and by the Institutional Ethics Committee (CE/564/11), 10 October 2011.

### 4.2. Cytological Samples

Anal cytological samples were collected from MSM attending the STI/HIV Unit, San Gallicano Dermatological Institute IRCCS, Rome for the Surveillance Program of Anal Intraepithelial Neoplasia (SAIN project [30]). Participants of the SAIN project included MSM aged ≥ 18 years who did not show clinically evident HPV-related ano-genital lesions. Anal samples were collected using a Dacron swab, dislodged in PreservCyt solution (Hologic Inc., San Diego, CA, USA). Liquid-based cytological slides were obtained using ThinPrep processor 2000 (Hologic Inc., CA, USA), and the morphology was evaluated according to the Bethesda system [31,32]. HPV testing (Linear Array HPV Genotyping Test; Roche Diagnostics, Indianapolis, IN, USA) was performed until resources were available, i.e., up to 2019. After performing HPV testing and cytology, the residual material was stored at 4 °C. Specimens adequate for morphological interpretation and HPV16 positive were retrospectively selected among those with residual PreservCyt sample sufficient to perform the investigations of interest for this study. Among the 69 samples with adequate cytology and HPV16 infection, collected between January 2016 and January 2019, 27 had enough residual PreservCyt sample. Among them, all HPV16-positive samples that could not be referred to high-grade lesions [9] LSILs, 5 atypical squamous cells of undermined significance (ASCUS), and 5 NILM samples] were named group A, and the remaining 8 samples, described as HSILs, were clustered in group B. For continuity of nomenclature, the total group of 27 HPV16 positive samples was called the A+B group in this text.

No reports of squamous cell carcinoma were found. Four randomly taken HPV16-negative anal samples were also included as negative controls.

All patients provided their informed written consent before participating in the SAIN study (CE/564/11).

### 4.3. Primers

Oligonucleotide primers necessary for target genes and the housekeeping gene were chosen by using the online tool Primer-BLAST and purchased from Invitrogen. The following primers were used: for the HPV16 E5 target gene, 5′-CGCTGCTTTTGTCTGTGTCT-3′ (sense), 5′-GCGTGCATGTGTATGTATTAAAAA-3′ (antisense); for the Snail1 target gene: 5′-GCTGCAGGACTCTAATCCAGA-3′ (sense), 5′-ATCTCCGGAGGTGGGATG-3′ (antisense); for the Snail2 target gene, 5′-TGGTTGCTTCAAGGACACAT-3′ (sense), 5′-GCAAATGCTCTGTTGCAGTG-3′ (antisense); for the FGFR2c target gene, 5′-TGAGGACGCTGGGGAATATACG-3′ (sense), 5′-TAGTCTGGGGAAGCTGTAATCTCCT-3′ (antisense); for the ZEB1 target gene: 5′-GGGAGGAGCAGTGAAAGAGA-3′ (sense), 5′-TTTCTTGCCCTTCCTTTCTG-3′ (antisense); for the TRPA1 target gene, 5′-TAATGGGAAAGCCACCCCTC-3′ (sense), 5′-GCACCTTCCCTTCTCCACTG-3′ (sense); for the 18S rRNA housekeeping gene, 5′-CGAGCCGCCTGGATACC-3′ (sense), 5′-CATGGCCTCAGTTCCGAAAA-3′ (antisense).

### 4.4. RNA Extraction and cDNA Synthesis

Cytological samples were centrifuged at 1500 rpm for 10 min, and the supernatant was discarded. The pellet thus obtained was resuspended in 500 µL of TRIzol (Invitrogen, Waltham, MA, USA); then, the total RNA was extracted following the protocol according to manufacturer’s instructions and eluted with 0.1% diethylpyrocarbonate (DEPC)-treated water. In order to purify and concentrate the extracted RNA, the eluate was subsequently re-extracted using the Quick-RNA™ Microprep Kit (Zymo Research, Irvine, CA, USA) by applying the standard protocol. The total RNA concentration was quantitated by the Nanodrop 1000 (Thermo Fisher Scientific, Waltham, MA, USA); 0.3–0.6 µg of total RNA was reverse transcribed using the iScriptTM cDNA Synthesis Kit (Bio-Rad, Hercules, CA, USA), with a mixture of oligo (dT) and random primers according to the manufacturer’s instructions.

### 4.5. PCR Amplification and Real-Time Quantitation

qRT-PCR was performed in triplicate with SYBR Green (Bio-Rad, Hercules, CA, USA), using 15 ng of cDNA, on a IQ5 real-time PCR detection system (Bio-Rad, Hercules, CA, USA). The reaction was carried out in a 96-well plate adding forward and reverse primers for each gene and 7 μL of diluted template cDNA to achieve a final reaction volume of 15 μL. All assays included a negative control and were replicated three times. The thermal cycling program was performed as described previously [9]. The data were analyzed using the 2^−ΔΔCt^ method. The results are reported as mean values ± SD from three different experiments in triplicate.

### 4.6. Statistical Analysis

Target gene expression was quantified in triplicate for each cytological sample from three independent experiments. Mean mRNA levels ± SD were visualized as bar graphs. Correlation and regression analyses (MedCalc version 19.5.3) were used to model the relationships between the gene expression levels. Scatter plots depicting these relationships were presented, showing the R^2^ Pearson correlation coefficient, the regression line ±95% CI, and the ±95% prediction bands. *p* values < 0.05 were assumed to be statistically significant.

### 4.7. Bioinformatical Analysis

The Biological General Repository for Interaction Datasets Biogrid database (https://thebiogrid.org (accessed on 10 June 2024))) contains an abundance of data on genetic and physical interactions, which can enable the discovery of relationships between genes and proteins, whether they have a demonstrated physical interaction or interactions based on inferences from the scientific literature [24]. BioGRID was queried to predict the proteins that could potentially interact with 16E5, and we used the “Network” module to create the protein interaction network of the 16E5 gene, with the image display set to “concentric circles”.

## Data Availability

Data is contained within the article.
